# A Strategy to Synthesize Multilayer Graphene in Arc-Discharge Plasma in a Semi-Opened Environment

**DOI:** 10.3390/ma12142279

**Published:** 2019-07-16

**Authors:** Hai Tan, Deguo Wang, Yanbao Guo

**Affiliations:** 1 College of Mechanical and Transportation Engineering, China University of Petroleum, Beijing 102249, China; 2 Beijing Key Laboratory of Process Fluid Filtration and Separation, Beijing 102249, China

**Keywords:** multilayer graphene, synthetic strategies, arc-discharge plasma, semi-opened environment

## Abstract

Graphene, as the earliest discovered two-dimensional (2D) material, possesses excellently physical and chemical properties. Vast synthetic strategies, including chemical vapor deposition, mechanical exfoliation, and chemical reduction, are proposed. In this paper, a method to synthesize multilayer graphene in a semi-opened environment is presented by introducing arc-discharge plasma technology. Compared with previous technologies, the toxic gases and hazardous chemical components are not generated in the whole process. The synthesized carbon materials were characterized by transmission electron microscopy, atomic force microscopy, X-ray diffraction, and Raman spectra technologies. The paper offers an idea to synthesize multilayer graphene in a semi-opened environment, which is a development to produce graphene with arc-discharge plasma.

## 1. Introduction

Since the graphene was isolated in 2004 by A.K. Geim and K.S. Novoselov via the ‘Scotch tape’ method [1], research on graphene has attracted a deluge of interests from scholars due to its extraordinary properties (excluding large available specific surface areas) and potential applications [2,3,4,5], especially in lithium ion batteries [5]. Graphene is the strongest discovered material in the world, whose elastic modulus reaches about 1.0 TPa [6]. In a word, graphene possesses a wide range of applications in our daily life and industrial manufacturing. 

After a slow start, there has been a rapid increase of the amount of research on graphene in recent years [7]. For example, less than 1000 patent applications were lodged before 2008. However, the number of patent publications lodged on graphene was more than 24,000 from 2008 to 2014. In these patent publications, various graphene synthetic methods have been presented. Current methods require an ultra-low pressure (vacuum in the quartz tube is always lower than 9.75 Torr) or toxic oxidation and reduction reagents [8,9,10,11]. Graphite oxide (GO) is the essential material for the oxidation-reduction method, and sometimes even for the atmospheric plasma method [12]. When synthesizing graphene by the oxidation-reduction method, strong oxidizing reagents with a pungent odor and strong causticity are barriers to operators [13,14]. Shahriary’s team [15] used a modified Hummers method to oxidize graphite powder, and harmful sulphuric acid (H_2_SO_4_) and potassium permanganate (KMnO_4_) were used. In addition, mechanical exfoliation of highly oriented pyrolytic graphite (HOPG) is a method that is suited to use in laboratory investigations due to its low yield [16,17]. Moreover, the cost of the epitaxial growth method is unacceptable in commercial production [18]. Chemical vapor deposition (CVD) is considered the most potential method to synthesize graphene, with high quality and large-scale in industrial manufacture. However, substrates (such as copper foils) are essential to be used to deposit and separate out the carbon atoms [19,20,21]. For further use, the graphene film should be transformed to the target material surface. The transformation processes—regardless of whether it is the traditional ‘wet etching method [22]’ or other advanced methods [23,24]—introduces defects on the surface of graphene. Lin et al. [25] indicated poly (methyl methacrylate) (PMMA) residues on the graphene surface as a barrier to decrease its performance, and the PMMA residues could only be decreased by the annealing process but could not be eliminated. Recently, Zhang et al. [26] reported a novel method to transform graphene without any polymer. The graphene was etched in the hexane and ammonium persulfate solution and then transformed to target substrates with the aid of a Si/SiO_2_ plate. This method depends on the quality of graphene. If the quality of graphene is good, the transformation process is not complex. Among these synthetic strategies, mechanical exfoliation gives the best graphene to date. Graphene synthesis by the epitaxial growth method is non-uniform in thickness and the chemical reduction introduces functionalized organic groups into this 2D structure. Nowadays, some new technologies, such as roll-to-roll technology [27], have been studied to enlarge the size of graphene film based on the CVD method. However, substrates with a special surface structure are expensive and suffer from time costs.

In 1990, fullerene (C_60_) was firstly synthesized using the direct current (DC) arc-discharge method by Kratschmer and Huffman with a high yield [28]. Moreover, Iijima and Toshinari [29] found single-shell carbon nanotubes in cathodic products when they used the arc-discharge method to synthesize fullerene. Arc-discharge plasma technology, with different sizes of graphite rods as the cathode and anode, is widely carried out to synthesize carbon nano-materials in a relatively confined space in a special atmosphere. Until now, multilayer graphene has been successfully synthesized by using this technology. Wu et al. [30] synthesized large-scaled few-layered graphene in the condition of carbon dioxide (CO_2_) and helium (He) with an optimized current (about 150 A) in a closed water-cooling stainless steel chamber. Chen et al. [31] synthesized graphene in a mixture of hydrogen (H_2_) and buffer gases at 400 Torr in a relatively short time (about 20 min). This proves that the arc-discharge method has the potential to quickly synthesize graphene. However, to our knowledge, a strictly experimental environment, such as whole ambient pressure, is needed in the synthetic process by arc-discharge plasma and the mechanism has seldom been discussed.

Herein, we will describe a novel methodology based on the plasma technique that can synthesize multilayer graphene in a semi-opened environment, which lowers the requirement of experimental conditions. A semi-opened environment means that there is no strict requirement of a sealed environment for the set-up. The local pressure around the two electrodes is maintained at 400 Torr. In addition, the method does not generate by-products of toxic gases or use hazardous chemical components, which are not friendly to operators. The synthesized material in the cathode and anode were characterized and analyzed with a number of techniques, including transmission electron microscopy (TEM), atomic force microscopy (AFM), Raman spectroscopy, and X-ray diffraction (XRD), respectively. The synthesized mechanism was further discussed depending on the dynamics of thermal plasma.

## 2. Experiment

### 2.1. Materials

**Figure 1:**
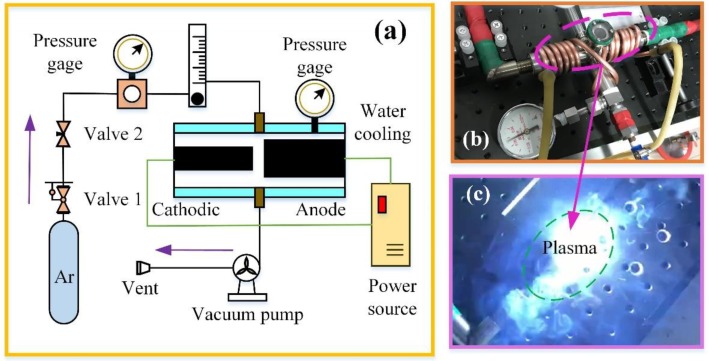
Arc-discharge plasma experimental set-up, (**a**) schematic diagram (**b**), real set-up, and (**c**) plasma generation area.

Graphene was directly synthesized without catalysts using two different sizes of graphite rods as carbon sources by arc-discharge plasma in a semi-opened environment. The graphite rods used in the experiment of 99.99%. A six-millimeter-diameter graphite rod in a length of 10 mm was employed as the cathode and the anode was also a 10 cm long graphite rod but in a diameter of 10 mm as shown in Figure 1b. Prior to experiment, the graphite rods were rinsed in a sonic washer with deionized (DI) water for 10 min and then dried in the nitrogen flow. Argon (Ar) with a purity of 99.999% was used in the experiment.

### 2.2. Experimental Set-Up

As shown in Figure 1, this experimental set-up mainly consists of an arc-reaction system, a pressure control and test system, and an auxiliary vacuum system. As we can see in Figure 1a, the cathodic rod and anodic rod were in the chamber and the distance of these two rods was about 2 mm. Arc plasma was generated between these two rods in the arc-reaction chamber (Figure 1c). In order to ensure the sustainability of the experiment, the chamber was cooled by water. The cathodic rod was grounded through a cable. A welder power source (WS-400, Beijing Time Technologies Co., Ltd), which was triggered in high frequency, was selected to ensure the stability of the arc ignition process. The welder power source offered a constant current of 35 A. The local ambient pressure around the arc-charge area was maintained at 400 Torr by a mechanical screw vacuum pump in the experimental processes. An Ar flow was directly blown to the arc-discharge area with a speed of 21.6 slm. The synthesized materials in the anode and cathode were collected for further characterization and analysis, respectively.

### 2.3. Characterizations 

Raman spectroscopy (NRS-3000, JASCO Co., Tokyo, Japan) is one of the effective characterization methods to identify the product. The excited wavelength was 532 nm. A laser beam was focused through a micro-scope objective with a high numerical aperture (100×) and NA was set as 0.9. The incident laser power was 1 mW to prevent the pristine sample from being modified. X-ray diffraction (XRD) was measured by a D8 Focus (Bruker Co., Inc., Karlsruhe, Germany) with monochromatized Cu/Kα radiation (λ = 1.5418 Å). The tube voltage and current were 40 kV and 40 mA, respectively. To further determine the product, transmission electron microscopy (TEM, Tecnai G2 F20, FEI, Hillsboro, America) and electron diffraction images were used to get the information of topological and layer number of the product (Tecnai G^2^ F20, FEI, operated at 200 kV). The thickness of the synthesized material was measured accurately in the tapping mode of an atomic force microscopy (AFM, Bruker Co., Inc., Karlsruhe, Germany).

## 3. Results and Discussions

**Figure 2:**
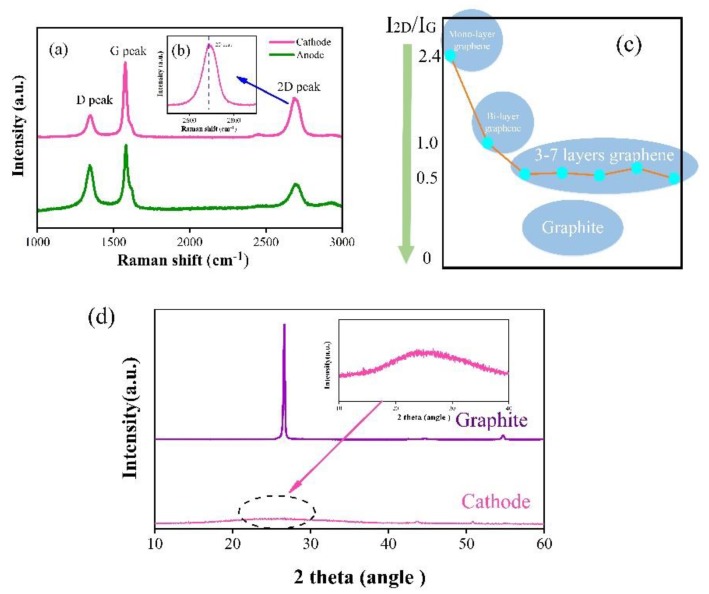
Raman spectra of the synthesized material (**a**) in different electrodes; (**b**) partial enlarged drawing of 2D peak (insert); (**c**) I_2D_/I_G_ intensity ratio schematic diagram; and (**d**) XRD patterns.

The samples for Raman spectroscopy analysis were sonicated in ethyl alcohol for 10 min and then the solution was dropped on an SiO_2_/Si wafer. Figure 2a shows the Raman spectra of synthesized material in different electrodes. The D-peak, 2D-peak, and G-peak were used to identify the synthesized materials’ type. For graphene, the D peak that stands for the activation by defects is around 1350 cm^−1^. Graphene with defects presents the existence of other bands and these bands are activated due to the breaking of the crystal symmetry that relax the Raman fundamental selection rule [32]. Such a phenomenon leads to an appearance of a D peak. The intensity of the 2D peak (~2700 cm^−1^), which is derived from inelastic scattering of two phonons, and the G peak (~1580 cm^−1^), which is related to the ordered in-plane sp^2^ carbon structure, can be used to distinguish the number of graphene layer (mono-, bi-, or few-) [33]. For the double resonance mechanism, in the peak around 2700 cm^−1^, only a resonant one can modify the Raman cross section. After the scattering process, the excited electron would be scattered back due to the phonon or a defect, and finally, a new photon is emitted [32]. As shown in Figure 2a, the synthesized material in the cathode (pink line) can be proven to be the graphene in a few layers according to Figure 2b,c. The shape and intensity of the 2D peak is sensitive to the number of graphene layers [34]. The insert partial enlarged drawing of the 2D peak (Figure 2b) appears to be symmetrical and the value of the symmetry axis is around 2690 cm^−1^ (a little bit less than 2700 cm^−1^). With the increase of the graphene layers (from a monolayer to multilayers), the 2D peak upshifted [35]. For graphite, the shape of the 2D peak, which can be considered as an assembly of two smaller peaks (2D_1_ and 2D_2_ peaks), is not symmetrical and the 2D peak exceeded 2700 cm^−1^ [33,36]. The intensity ratio of 2D and G peaks of the synthesized products in the cathode is about 0.52. Tu and his team confirmed that the layer of graphene changes from three to seven layers when the ratio is around 0.5 as shown in Figure 2c [37]. It can also be found in Figure 2a that the synthesized material in the anode (green line) is the graphite in little layers due to the I_2D_/I_G_ intensity ratio being less than 0.5. The I_D_/I_G_ intensity ratio is an assessment method of the defect level. Compared with the cathode, the I_D_/I_G_ ratio in the anode is greater than 0.7, thus showing more defects. 

Figure 2d shows the XRD patterns of the material in the cathode and the material collected from the original graphite rod. The strong peak in the XRD pattern of the material collected from the original graphite rod appears at 26.6°. For the material deposited in the cathode, there are weak and broad peaks at 24.8°, similar to [38].

**Figure 3:**
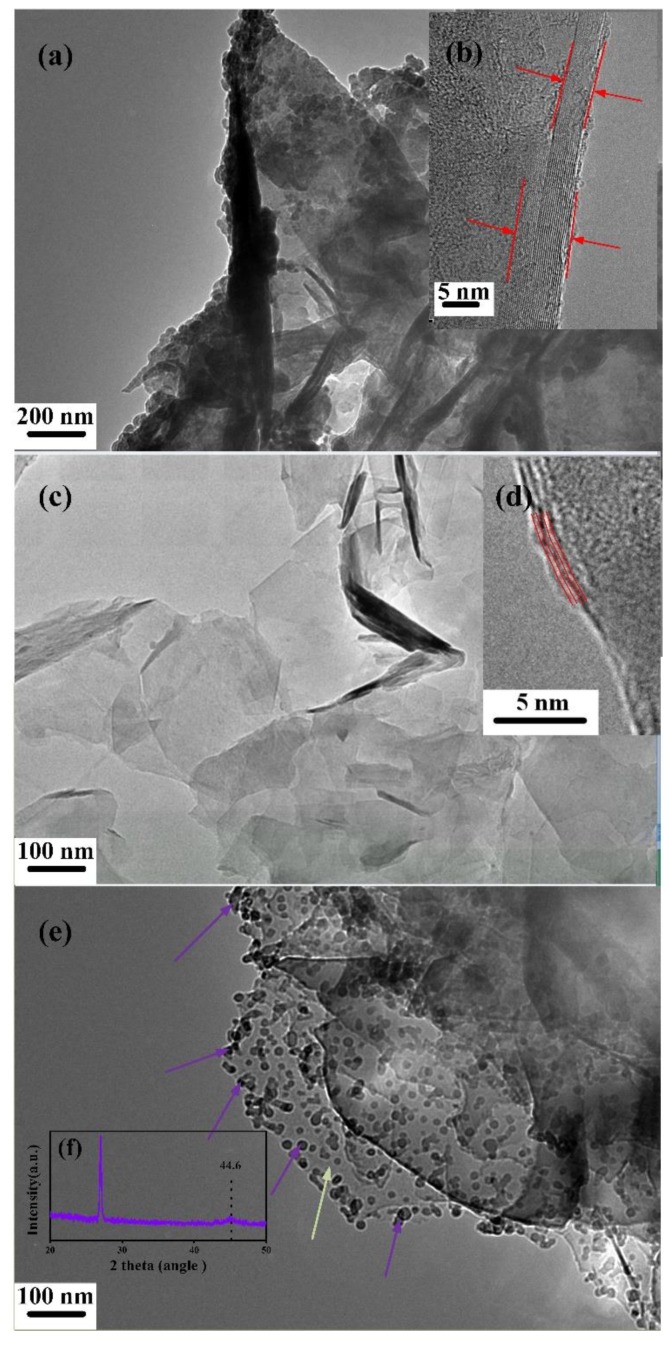
Transmission electron microscopies of (**a**) graphite at low magnification, (**b**) graphite at how magnification, (**c**) synthesized material in the cathode at low magnification, (**d**) synthesized material in the cathode at high magnification, (**e**) synthesized material in the anode, and (**f**) XRD of synthesized material in the anode.

**Figure 4:**
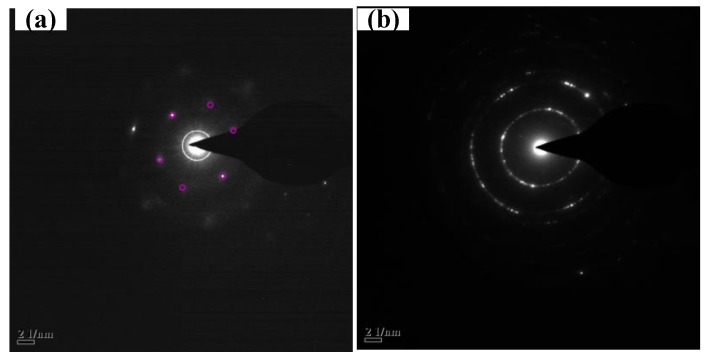
Electron diffraction images of (**a**) synthesized material in the cathode and (**b**) synthesized material in the anode.

To further identify the materials synthesized in the cathode and anode, the TEM and electron diffraction images were employed. The samples were first sonicated in dimethyl formamide (DMF) for 2 h, and then centrifuged. After that, the supernatant liquid was dropped on the micro-grid and dried by an infrared lamp. The information of the graphite and synthesized materials were detected by TEM as shown in Figure 3. The synthesized material in the cathode (Figure 3c,d) was more transparent and thinner after the arc-discharge process compared with the original graphite (Figure 3a,b). The layer information can be observed clearly in the high magnification TEM images as shown in Figure 3b,d. The TEM image of the graphite (Figure 3a) showed a low level of transparency compared with the synthesized materials’ TEM images. Additionally, the layer of graphite is more than 18 according to Figure 3b. It can be concluded from Figure 3d that the graphene synthesized in the cathode is four layers, and this conclusion is consistent with the Raman analysis presented above. The corresponding electron diffraction (ED) pattern of the synthesized material in the cathode is shown in Figure 4a. The carbon atoms are arranged in the hexagonal crystal mode marked by the purple circles. The TEM information proves that the graphene in a few layers has been successfully synthesized on the cathode.

Compared with the material synthesized in the cathode, the product deposited on the anode possessed a high defect as mentioned above. Figure 3e and Figure 4b show the TEM and ED images of the material synthesized in the anode. We can see in Figure 3e that there are many black dots (pointed by the purple arrows) in the thin film (pointed by the green arrow). These black dots are expected to result in a sharp increase of the I_D_/I_G_ ratio in the anode. To identify the composition of the material synthesized in the anode, XRD was employed. We found a slight peak around 44.6° in Figure 3f. This showed that Fe element was present. Beside the metal Fe, a sharp XRD peak was remarkably found at about 26.6° corresponding to the crystal structure of graphite according to the JCPDS card (No. 41-1487). The ED image of the synthesized material in the anode shows a more unordered arrangement. This phenomenon is also consistent with the TEM image.

**Figure 5:**
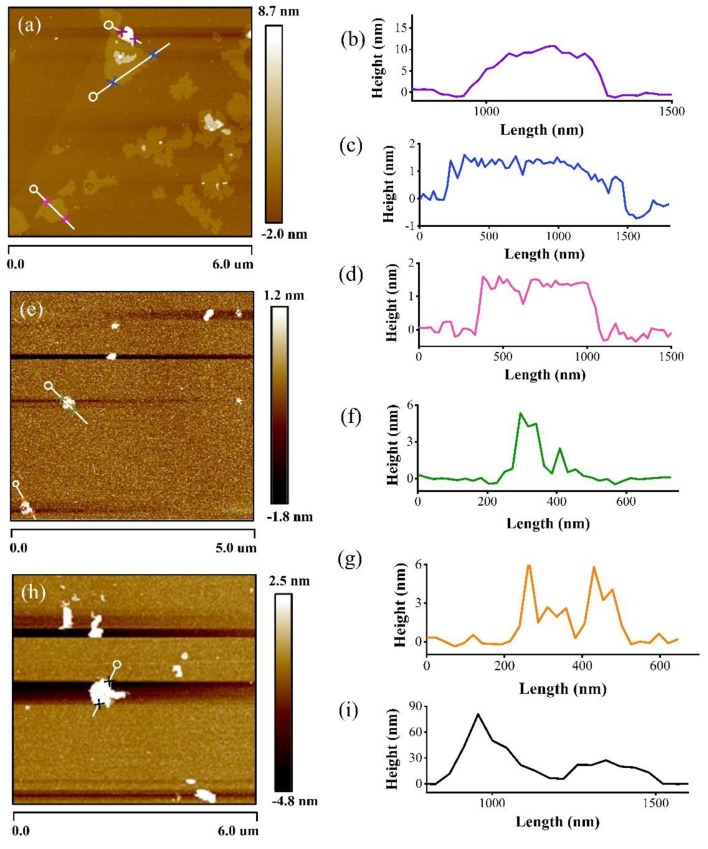
AFM (Atomic force microscopy) images of synthesized material in the cathode on mica surface, (**a**) AFM image (**b**–**d**) its height profiles, the material in the anode; (**e**) AFM image, (**f**,**g**) its height profiles, and the material collected from original graphite rod; (**h**) AFM image and (**i**) its height profile.

The synthesized products in the cathode and anode were sonicated in the ethyl alcohol, respectively. Then, they were dropped on the mica surface for the AFM test to obtain the AFM images and the thickness information. The AFM testing of material collected from the original graphite rod was also detected. It can be seen that from the AFM image (Figure 5a,b) that the height of the brightest area is more than 8 nm, showing that the material in this area is not graphene, but is graphite. The carbon atoms were accumulated in this area that lead to the increase of the thickness. However, for most of the region in Figure 5a, the height is lower than 2 nm (Figure 5c,d). This indicates that the graphene in a few layers had been synthesized. The average particle size is about 1.0 μm. The AFM image and its height profiles of the material synthesized in the anode are shown in Figure 5e–g. It is obvious that the height of the anode material (about 6 nm) is much higher than the cathode due to the defects in the material as shown before. Figure 5h,i show the AFM and height profile of the material collected from the original graphite rod. The height is much higher than the synthesized materials. After the whole process of characterization and analysis, we can say that the graphene in a few layers has been successfully synthesized in the cathode. Moreover, the products deposited on the anode were a few layer graphite with Fe element.

**Figure 6:**
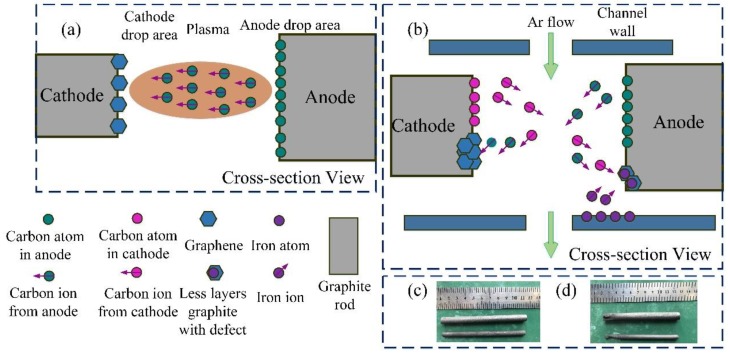
Synthetic mechanism of graphene (**a**) without Ar flow, (**b**) with Ar flow, and macro morphologies (**c**) before (**d**) after arc-discharge.

Now, the growth mechanism of graphene in the arc-discharge plasma condition is discussed. The growth pattern for synthesizing graphene without substrate is shown in Figure 6. Individual carbon atoms should be evaporated and then re-nucleated in the structure of a regular hexagon as shown in Figure 5a when the temperature between the anode and cathode electrodes is bigger than 3500 K. Figure 6a,b indicate the synthesized mechanism when using arc-discharge plasma technology without and with Ar flow, respectively. The type of synthesized carbon nanomaterial is limited by the temperature. For example, Keidar et al. [39] obtained single-wall carbon nanotube (SWNT) in the temperature range of about 1200–1800 K. When the gas between cathode and anode is triggered by high frequency and arc-discharge occurs, the temperature is more than 3500 K [40]. This high temperature lead to the evaporation of the carbon atom in the anode (Figure 6a). The carbon atom further changed to a carbon ion as shown in Equation (1). The graphene synthesized in the cathode in a special atmosphere as Chen et al. discussed [31]. The heat dissipation was achieved by the molecular heat conduction mechanism without gas flow in the chamber. Thus, the cathode is regarded as a deposited medium and graphene powder can be collected on the cathode. It is interesting that the different synthesized material grows in the cathode and anode in this experiment that is mentioned above. It can be seen in Figure 6d that the graphite rods of both the cathode and anode were consumed. Before the arc-discharge process, the graphite rods are complete and dense as shown in Figure 6c. However, after the experiment, the graphite rods become more porous and lots of hole can be seen through the eyes. When the Ar flow is introduced into the system, part of the heat may be taken away by Ar flow and bring the oscillation between the anode and cathode. Part of the arc column forms a closed system with the channel wall and thus some iron atoms evaporate to form iron ions (Equation (2)). These iron ions combine with carbon ions to nucleate and grow to the doped-graphite as shown in Figure 6b.

(1) $$C+U_{i}\overset{Arc}{\to }C^{+}+e$$

(2) $$Fe+U_{i}\overset{Arc}{\to }Fe^{+}+e$$

where *C* and *Fe* are the carbon atom, *U_i_* is the foreign energy, *Arc* represents the changing condition, and *C^+^* and *Fe^+^* are the carbon ion and iron ion, respectively.

## 4. Conclusions

Multilayer graphene was successfully synthesized in the cathode in a semi-opened environment by introducing arc-discharge plasma. The synthesized material deposited on the cathode and anode was characterized and analyzed by Raman spectrum, TEM, XRD, and AFM. These results show that the graphene in a few layers was synthesized. Compared with the synthesized material in the cathode, the product in the anode showed a different structure. As we can see in the TEM images, the products deposited on the anode possess many black dots in the thin film, which is the reason why the I_D_/I_G_ ratio (Raman spectrum) of the anode is high with a graphitic structure. Moreover, the synthesized mechanism was further discussed depending on the dynamics of the thermal plasma. When the gas between the cathode and anode was triggered and arc-discharge occurred, individual carbon atoms should evaporate from both the anode and cathode and then re-nucleate in the structure of a regular hexagon. In summary, we provided a method to synthesize multilayer graphene in a semi-opened environment by introducing arc-plasma technology.
